# Challenges of Implementing an Effective Primary Health Care Accreditation Program: a qualitative study in Iran

**DOI:** 10.1186/s12875-023-02232-0

**Published:** 2023-12-13

**Authors:** Farid Gharibi, Esmaeil Moshiri, Masoumeh Ebrahimi Tavani, Koustuv Dalal

**Affiliations:** 1 Social Determinants of Health Research Center, Semnan University of Medical Sciences, Semnan, Iran; 2 Academic Research Staff, Quality Improvement, Monitoring and Evaluation Department, Center of Health Network Management, Deputy of Public Health, Ministry of Health and Medical Education, Tehran, Iran; 3 Division of Public Health Science, Department of health Sciences, Mid Sweden University, Sweden and Institute for Health Sciences, University of Skovde, Skovde, Sweden

**Keywords:** Public Health, Primary Health Care, Quality, Accreditation

## Abstract

**Background:**

Accreditation is a prerequisite for scientific management of the health system, owing to its numerous benefits on health centres’ performance. The current study examined Iran’s primary healthcare accreditation program to ascertain the challenges to its successful implementation.

**Methods:**

This qualitative study examined the perspectives of 32 managers and staff members in the pilot accreditation program (from the Ministry of Health and Medical Education, Semnan University of Medical Sciences, and Aradan District Health Network). Three in-depth group interviews were conducted using a semi-structured questionnaire, and the data obtained were assessed using thematic analysis. As a result of this investigation identified six themes, 29 sub-themes, and 218 codes as challenges to the successful accreditation of primary health care in Iran.

**Results:**

Six main themes, including “organisational culture”, “motivational mechanisms”, “staff workload”, “training system”, “information systems”, and “macro-executive infrastructure”, were identified as the main domain of challenges, with seven, five, two, four, three, and eight sub-themes respectively.

**Conclusion:**

Accreditation of PHC in Iran faces significant challenges and obstacles that, if ignored, can jeopardise the program’s success and effectiveness. By identifying challenges and obstacles and making practical suggestions for overcoming them, the findings of this study can aid in the program’s successful implementation and achievement of desired outcomes.

## Introduction

Primary health care (PHC) provides comprehensive, equitable and cost-effective health-related services to the covered population in their living place, such as home, school, and workplace. Although the PHC delivers basic therapeutic care, it emphasises preventive and promotional services [1]. Due to the vital role of PHC in procuring community health and well-being, continued quality improvement in their related fields has long been a primary concern for international health-related organisations, health systems, insurance companies, and care recipients [2].

Accreditation is a practical approach to enhancing the performance and quality of services provided by health facilities, especially the PHC health centres [3]. Accreditation is how healthcare providers demonstrate their competency in delivering high-quality healthcare by complying with the requirements outlined in the accreditation standards [4]. The primary contrast between accreditation and other types of evaluation, such as licensure and certification, is in the standards and criteria utilised for accreditation. While other systems emphasise meeting minimum and input requirements, accreditation fosters quality improvement in health care by setting demanding and maximum standards and stressing expertise through process and output indicators [5].

“The accreditation programs affect the PHC health centers performance positively in a wide range of health system performance domains including quality, effectiveness, human resources management, safety, customer satisfaction, responsiveness, documentation, strategic management, leadership, accessibility, efficiency and continuity of care” [3]. Naturally, achieving a wide range of positive outcomes through accreditation, as well as their sustainability, is dependent on several factors, including the quality of standards and evaluation criteria, the quality of the accreditation process, evaluators’ professional competence, and the development of individual and organisational motivation systems [6].

After one century of emerging hospital accreditation models, the PHC accreditation models were developed in the recent decade by the Joint Commission International [5]. Several countries, particularly in the Eastern Mediterranean region, such as Jordan, Lebanon, Egypt, Qatar, and Saudi Arabia, commenced accreditation practice with the assistance of pioneers, the United States and Canada, and consultation of the International Society for Quality in Health Care (ISQua) [7]. Unlike hospital accreditation models, which are emphasized focal areas such as patient assessment, safety and infection control, and medication management, the focal area of PHC accreditation models is emphasized on community-orienting, accessibility of care, continuity of care, and internal-external collaboration due to their mission and aims [8].

Although the ISQua published more than 100 years of world experience in the accreditation [9], assessing challenges and shortcomings from the users’ viewpoints of an accreditation model in any field is very important [10]. In 2018, Iran created and disseminated a national model for functional accreditation of the PHC centres. This model defines 12 functional areas based on forward-thinking and successful worldwide models, the most recent valid scientific facts, most notably the ISQua standards, and the opinions of all domestic experts [7]. Accreditation standards and metrics are defined under these functional domains [8]. Considering the designers’ emphasis and the widespread tendency in similar situations that necessitate the execution of pilot accreditation models before scaling up the program, in 2020, the Ministry of Health attempts to identify the program’s challenges and related development methods. Therefore, Aradan’s health network at Semnan University of Medical Sciences was selected as the program’s pilot plan, and the pilot test was conducted in 2 years [11]. The current study aimed to identify and depict the challenges of implementing an efficient accreditation program in primary health care in Iran as perceived through the eyes of model users.

## Materials and methods

The current study was conducted in 2021 using a qualitative method. Through three group interviews, we obtained the viewpoints of 32 managers and staff members participating in the pilot program. Like other qualitative studies, the initial participants of the study were selected through available sampling, and the point of view of all participants was used in the pilot of the accreditation program. In the following, purpose-based sampling was used, which means that there was a particular emphasis on obtaining and benefiting from the opinions of more informed participants [12]. The pilot program was designed so that the managers and staff members of the Ministry of Health and Medical Education, Semnan University of Medical Sciences’ health office and the Aradan District Health Network were interviewed separately, with 7, 10 and 15 interviewees, respectively. Intervention and promotion programs were developed and implemented following independent groups’ initial self-assessment and external evaluation. Finally, a secondary evaluation was carried out as a self-assessment and an external evaluation. The success of the treatments was determined by comparing primary and secondary evaluation data. According to the findings of this study, program participants believe it is vital to tackle the challenges and difficulties of having a successful accreditation program.

The interviews were performed using a semi-structured questionnaire with open-ended questions concerning the participants’ perceived challenges, obstacles and shortcomings to prevent bias in the participants’ opinions (such as; “What are the challenges/obstacles/shortcomings in the successful implementation of Iran’s PHC accreditation program?”). The questions were determined by getting consultations with the accreditation field’s experts. The interviews were 75, 95, and 120 minutes long, respectively (97 minutes average). With the participants’ agreement, all the interview sessions were taped. The interviewers also noted their thoughts on the perspectives offered and provided comments to the interviewee to ensure their intention was accurately understood. During the interview, the emphasis was on all respondents’ explicit and free expression. Employee interviews were conducted separately from manager interviews. The management of the interview sessions was done in such a way that certain participants did not dominate the others. This was done through the timing of the comments and the participation of each participant in the discussions. All interviews were conducted until the information saturation stage, when all thoughts and points of view were expressed, and the participants addressed no new topics.

The thematic analysis approach was employed to analyse the interview data because it enables the systematic extraction, categorisation, and reporting of qualitative findings [12]. The recorded contents of the interviews were transcribed word by word and supplemented and validated with the collected manuscripts. Then, the significant components that mentioned participants’ perceived obstacles and challenges were identified and established as codes by two coders using an open coding approach. Suppose any discrepancy has arisen between the coders resolved by discussion and collaboration with other researchers. Following that, similar codes were combined, resulting in sub-themes and, eventually, main themes. Finally, six themes, 29 sub-themes, and 218 codes were identified. The study’s rigour (reliability of findings) was enhanced through several methods, including providing interviewees with feedback on their responses during the interview, simultaneous and independent analysis of data by authors, and comparing and integrating the results of independent analysis throughout the sessions in collaboration with qualitative experts [12]. Researchers adhered to ethical principles, including obtaining approval from the university’s ethics committee, giving participants complete freedom to participate or withdraw from the study, obtaining informed and written consent from participants, and assuring them that their views would be undisclosed. Additionally, the authors will prioritise the exclusive use of the study results.

## Results

The current study identified six major themes: organisational culture, motivational mechanisms, staff workload, training system, information systems, and macro-infrastructure, each containing seven, five, two, four, three, and eight sub-themes, respectively. These sub-themes stand out as the 29 main impediments to the successful implementation of the accreditation program (Table 1).

**Table 1:** The challenges to successfully implementing Iran’s PHC accreditation program

Category	Challenges and shortcomings
**Organisational culture**	The system’s quantitative nature and its conflicts with the implementation of quality improvement initiatives such as accreditation
	Considering accreditation as a temporary endeavour and its failure
	The negative impact of ineffective implementation of other promotional programs
	There needs to be more awareness of the importance of accreditation programs as an essential component in improving performance.
	Lack of persistence in implementing and attaining the program’s goals
	Doctors steadfastly refused to participate in the program despite their leadership positions at the facilities.
	An inadequate organisational culture of transparency and objective accountability
**motivational mechanisms**	Deficiency in defining the financial, professional, scientific, and spiritual motivations of the program for the participants
	Inadequate specification of organisational incentives of the program, such as budget and insurance tariffs for successful centres
	Employees and managers’ extreme reliance on external motives and the lack of continuity of these motivators
	Some centre workers, particularly the doctors, are on a temporary contract.
	Migration of skilled forces from less privileged places to affluent communities
**Staff workload**	A need for more personnel forces employees to take on various duties and responsibilities.
	Due to crises, the COVID-19 pandemic, and the high volume of health programs, employees are under great stress.
**Educational system**	Training for managers and staff on accreditation and quality improvement needs to be improved at the university level, as pre-service and in-service training.
	Accreditation programs in the country need more comprehensive and practical training sets.
	Inadequate knowledge of managers and staff of the studies on health systems
	System managers and family doctors lack the knowledge and abilities to administer the healthcare system effectively.
**Information system**	Inefficient design of the SIB system as a health information system
	Inaccurate data submission in SIB results in incorrect output.
	Lack of a verification method to ensure the accuracy of the submitted data in the SIB system, as well as other performance reports
**Macro-executive infrastructure**	The Deputy of the Ministry of Health and the University of Medical Sciences, as well as the country’s health networks, lack accreditation and a quality improvement unit
	More health management specialists and experts are needed to operate in the health care system.
	Lack of participation of international accreditation and quality improvement organisations in the design or implementation of the program
	Nationally, there is a deficiency in the training of accreditation assessors.
	Failure to appoint a competent and non-governmental entity to oversee accreditation with the cooperation of representatives from all relevant parties
	Lack of a clearly defined accreditation procedure in Iran
	Excessive system centralisation and a lack of environmental unit authority in the design and execution of health interventions
	Failure to approve the program in the form of a law governing its retention in the system

### Organisational culture

#### The system’s quantitative nature and its conflicts with the implementation of quality improvement initiatives such as accreditation

The system’s traditional focus on quantitative indicators related to health care evaluation and the design of existing mechanisms and systems based on them is one of the most significant organisational culture barriers to institutionalising programs related to improving health care quality, particularly the accreditation program. In such a system, all people involved in evaluation and accreditation, including managers, evaluators, and evaluatees, are based on the number of health services provided while ignoring the qualitative dimensions of care and even lacking the knowledge and experience to evaluate and promote them.

*“Quantitative perspectives are prevalent in PHC evaluation systems, consequalntly the evaluation indicators are typically quantitative. Individuals who conduct enough care are rewarded, regardless of the quality of care provided, the outcomes of care, or the level of satisfaction of service recipients.”* (Interview 3, Participants 9).

#### Considering accreditation as a transient Endeavour and its failure

The belief that accreditation is temporary research or executive initiative requiring health workers to be taught, gain expertise, or meet the program’s objectives is a critical cultural barrier to accreditation implementation in health facilities.

*“Previously, quality improvement programs centred on documenting and improving processes through the FOCUS-PDCA technique widely deployed at various levels in PHC. However, with the changing of the program’s founders at the Ministry of Health, everything was forgotten.”* (Interview 2, Participants 1).

#### Negative impact of ineffective implementation of other promotional programs

The temporary and sometimes ineffective implementation of similar quality improvement programs in recent years in hospitals and PHC has contributed to pessimism and significant opposition among healthcare employees to quality improvement programs.

*“Failure in programs such as quality-based payments in PHC in recent years fosters pessimism about quality improvement programs. The quality improving initatives are implements feebly”* (Interview 3, Participants 13).

#### Inadequate knowledge of the critical nature of accreditation programs as an essential part of performance improvement

Creating a culture recognising the value and necessity of utilising accreditation’s unique skills to improve healthcare quality is a significant task. Managers and health personnel must first acknowledge that significant problems exist in the performance of health centres and units under their control, particularly concerning the quality and safety of services, which can jeopardise their ability to fulfil their mission and goals. They must then understand the role of accreditation in resolving quality improvement issues by implementing required training programs and providing successful services.

*“The health providers constantly involved in their daily duties, not to improvement. They are satisfied with using only a few superficial and quantitative indicators to assess their performance. Evaluating health centres using accreditation standards and metrics, particularly in terms of process and outcome, can provide a clear picture of their performance and quality of care and help explain the need for accreditation.”* (Interview 1, Participant 4).

#### A lack of persistence in implementing and achieving the program’s objectives

Accreditation programs need significant effort and time to implement because the suggested model covers all functional dimensions and provides comprehensive standards and metrics for evaluating health centres. Such a program’s comprehensive and effective implementation necessitates careful planning and uninterrupted and ongoing efforts over several years. Therefore the accreditation program necessitates capacity building and a fundamental shift in the perspectives and attitudes of health centre management and staff.

*“Adopting a complete accreditation program necessitates a great deal of patience, which managers and health workers at various levels do not now have. Essentially, it takes longer for interventions to be pushed and established to develop organisational culture.”* (Interview 2, Participant 6).

#### Physician reluctance to participate in the accreditation program, despite their managerial role in the centres

While physicians in PHC frequently serve as managers of health facilities in addition to their clinical responsibilities, they are reluctant to involve in improvement programs, particularly accreditation. This could result from deficiencies in instructional, motivational, and supervisory systems. It is worth noting that physicians’ non-compliance or poor compliance with the accreditation program can have adverse impacts. The physicians practising at the PHC care are considered the focal point of activity in these centres and play a critical role in motivating and assisting other staff.

*“Physicians are reluctant to engage in the accreditation program. Even their higher-level managers cannot force them to fulfil their accreditation duties.”; “The resistance and lack of motivation of health centre physicians are transferred to the down-line health workers, which can make it difficult to implement the accreditation program.”* (Interview 2, Participant 8).

#### Weakness in the organisation’s culture of transparency and objective accountability

Accreditation programs cannot be implemented successfully without establishing a solid organisational culture, particularly data-driven decision-making. It is vital to represent the performance of individuals, work units, and health centres fully and adequately in connection to the duties and programs assigned during the tenure and using transparent health indicators. In this regard, it must generate valid and reliable data and information and periodically analyse and approve their accuracy and reliability. One can only hope that personnel, work units, and health centres will be held accountable for their performance and efforts to improve it in such a situation.

*“The health system lacks a strong and widespread culture of functional transparency, as relevant performance indicators have not been developed. This defect is immediately apparent in corporate culture and may result in increased resistance to the accreditation program by low-performing individuals, centres, and units.”* (Interview 2, Participant 7).

### Motivational mechanisms

#### Deficiency in defining the financial, professional, scientific, and spiritual motivations of the program for the participants

Providing practical and consistent motivational mechanisms is a critical component of health program effectiveness, as it encourages individuals involved to make reasonable and persistent efforts to accomplish the goals. These motives should be taken seriously, not just on a financial level but also on a professional, scientific, and spiritual level. According to the findings of this study, a precise system for implementing accreditation programs has yet to be identified. It is worth noting that in other health initiatives, the motivators are frequently presented in an insufficient and unsustainable financial context.

*“The payments should be distributed fairly, with a greater share going to those who work harder or produce higher-quality work. The results of accreditation assessments of employee performance must be linked to payment and other reward systems and serve as the foundation for compensation and career advancement.”* (Interview 3, Participants 12).

#### Lack of a description of organisational incentives for successful centres in the program, such as budget and insurance tariffs

It is vital to consider special incentives for accredited centres and units along with individual motivations. For instance, successful centres can be awarded additional funds and equipment to provide better health care. Insurance companies may also consider charging lower rates for services and care offered by accredited organisations or prioritising them for funding and financial aid.

*“The link between accreditation outcomes and compensation granted to organisations participating in accreditation programs is important. It is necessary to distinguish between a centre that has expended a significant amount of time and effort to implement the accreditation program and improve its performance with a centre that has not had much success in this regard.”* (Interview 2, Participants 2).

#### Over-reliance on external motivations by personnel and management, despite their inconsistency

However, excessive reliance on external motivations can be detrimental because managerial changes can influence their consistency during economic downturns. In such a case, the program will be ineffective, and its achievements will be swiftly forgotten. Therefore, determine ongoing external motivators and certify the complete accreditation scheme in legislation with sustainable resources.

*“Politicisation of health care is a significant issue because it jeopardises the viability of incentive mechanisms. The health professionals and management have gotten accustomed to earning remuneration in addition to their normal salary for executing each new health plan. It is their responsibility to carry out any new program without regard for reward.”* (Interview 2, Participants 10).

#### Some Centre employees, particularly doctors, are temporary

The system’s lack of permanent employees can impede the successfully implementing of quality improvement programs, particularly accreditation. This is critical from two perspectives. First, temporary personnel (trainees and, to a lesser extent, corporate and contractual) will not be required to make the necessary effort to implement the accreditation program and upgrade the system since they will have a minimal obligation to existing centres and health systems. Second, after getting the necessary training and acquiring the requisite skills to meet the accreditation program’s requirements, this personnel will either be dismissed or transferred to other centres, resulting in a waste of training resources. Moreover, the health system will incur increasing costs in finding and replacing them.

*“Temporary staff, leave the system shortly after their assignment ends, and staff lacking accreditation knowledge and experience enter the system. Because the goal of these staff is to pass the mandatory plan and earn a portion of their income through participation in specialised clinical courses, They never exert sufficient effort and support to implement the accreditation program.”* (Interview 3, Participants 10).

#### The relocation of healthcare workers from low-income areas to more affluent places

The more fundamental problem is that because wealthier places such as capital cities or provincial capitals provide more options, health professionals, particularly non-natives, migrate to these cities to seek better opportunities. Permanent health staff may leave their position after 5 or 10 years of service. As a result, underserved areas will always struggle to attract and retain qualified health professionals who hire only temporary or inexperienced staff and incur additional costs due to the additional training required for new health workers.

*“Low-income communities, mainly rural areas and even small towns, lack adequate facilities and attractions. Additionally, health workers are less inclined to live and work in underprivileged areas as their basic needs and expectations are not fulfilled in these locations.”* (Interview 3, Participants 5).

### Employee workload

#### Employees’ multiple responsibilities and occupations due to a lack of manpower

Iran’s PHC system is highly comprehensive, and it addresses practically all public health issues to varying degrees and in various geographical areas. In such a case, a lack of staff results in their multi-occupancy, which lowers their time to participate in promotional activities such as accreditation program implementation. In such conditions, if the health personnel lack sufficient competence, experience, and knowledge to do their jobs effectively, the lack of time becomes much more intense.

*“Iran’s PHC system includes 12 distinct categories for providing services to different citizens. Every health worker is occasionally tasked with handling multiple critical sectors, leaving little time for accreditation and quality improvement activities.”* (Interview 3, Participants 1).

#### Employees are under tremendous stress due to crises, the COVID-19 pandemic, and the high volume of health programs

Numerous disasters, notably earthquakes and floods in Iran, have always burdened the health system because even the slightest carelessness during a crisis can result in widespread and catastrophic epidemics. Additionally, the outbreak of COVID-19 is notable in this aspect, as it is draining the funds of the health system. Due to the endless number of health workers, the pressure put on them by this epidemic has caused even some of their primary health responsibilities to be diminished and given less priority than programs related to this epidemic. Accreditation programs will face a severe challenge in these circumstances. Another significant point is that the availability or absence of adequate human resources in a health facility should be included in the accreditation findings and the final certification. They are expected to comply with the accreditation standards. There is complete equality in accreditation requirements and criteria.

*“Effective management of a crisis is require more resources especially human rreources. With certainty, the demand on staff has more than doubled since the Covid-19 pandemic, while the number of health professionals has remained nearly unchanged.”* (Interview 3, Participants 11).

### Training system

#### Training for managers and staff on accreditation and quality improvement is lacking at the university level, as well as pre-service and in-service courses

While implementing quality improvement programs, particularly accreditation and related activities requires purposeful and continuous teamwork with the conscious and wise participation of all health workers, managers, and health forces, particularly doctors working in care centres, which are unfortunately lacking in this regard. Primary healthcare workers receive no instruction during their university courses in this area. This significant deficit contributes to the health forces’ perception of no need for an accreditation program and adversely impacts their attention and efforts in this area. Another factor to consider is that these individuals receive no training when working in the health system or in-service programs.

*“Doctors and health workers have received essential training at the university to perform their job duties only. In such a situation, one cannot expect adequate knowledge, attitude, skills and behavior from health workers in the accreditation and quality improvement field.”* (Interview 2, Participant 5).

#### Accreditation programs in Iran are lacking in comprehensive and practical training

One of the most significant deficiencies is a scarcity of scientific and instructional materials for managers and healthcare employees in accreditation and quality improvement. The books and resources developed in this area, drawing on authentic and trustworthy international experiences, must consider the conditions and realities of Iran’s PHC system in educational courses. Along with the deficiencies in managerial knowledge, attitude, and abilities required for accreditation, health managers and staff also need to improve in implementing the requirements outlined in accreditation standards and criteria in the health system.

*“Providing a comprehensive set of training materials in the form of textbooks and manuals for accreditation-related activities, can be helpful. Along with focusing on successful worldwide experiences and solid scientific facts, it is critical to ensure that this book is applicable and understandable to all managers and health workers.”* (Interview 1, Participant 6).

#### Inadequate knowledge of managers and staff of the studies on health systems

Research in the health system is critical for determining the extent and depth of systemic deficiencies, developing improvement solutions based on international experiences and the perspectives of domestic experts and process owners, and monitoring and evaluating intervention implementation and effectiveness. According to studies, a critical field of health system research in Iran’s primary healthcare system needs to be addressed. Few studies are conducted only by researchers at medical universities for their interests and ambitions, not for the system’s requirements.

*“In a system where its employees and managers lack the expertise even in assessing a status and identifying existing shortcomings, determinings and implementation of solutions, as well as reviewing the level of effectiveness of interventions, the activities conducte in a subjective manner and without regard to scientific evidence and reliable information.”* (Interview 2, Participant 6).

#### System managers and family doctors lack the knowledge and abilities necessary to administer the health care system effectively

While health system administrators must possess the necessary knowledge and expertise, graduates in healthcare administration, health policy, and health economics are frequently not placed in managerial roles. Additionally, the health system managers sometimes receive no management training, even at the most basic level, whether in a university setting or as a pre-or in-service employee. Another factor to consider is that general practitioners are appointed as directors of comprehensive health service centres immediately upon graduation from university, despite their lack of administrative and executive expertise.

*“The PHC managers are select based on inappropriate criteria. The reality is that there is no way to justify the employment of general practitioners without managerial experience as family physicians, as the health centers managers. It is a waste of financial, human, and educational resources.”* (Interview 2, Participants 4).

### Information systems

#### Inefficient design of the SIB system as a health information system

An efficient health system’s primary characteristics are purposeful design, appropriate and regular completion, proper maintenance, and proper use of care documentation. As a result, developing an effective information system for administrative and clinical use in health systems, particularly primary care, is vital and must be prioritised. Managers and personnel in PHC believe that the SIB system has serious flaws, particularly in computing all relevant or required metrics for evaluating health care. On the other hand, due to their preliminary design, statistical panels associated with health units and centres cannot provide a comprehensive and objective image of the service.

*“The SIB system, which is employed as a clinical and management information system in Iran’s PHC system, has an inferior design and is incapable of calculating many essential indicators of various dimensions of health care.”* (Interview 1, Participant 7).

#### Inaccurate data submission in SIB results in incorrect output

According to studies, completing various care components in the SIB system is highly time-consuming due to redundant components. Rather than evaluating and giving services to care recipients, health personnel are entirely focused on completing the system’s numerous sections. The noteworthy aspect in this regard is the quantitative nature of reward systems and their relationship to the completion of various components of the system, which results in employees reporting erroneous care and services, i.e., services that are never supplied to care receivers.

*“The SIB system defines multiple components for each care, which comprises numerous components that take time to complete and the system cannot provide all the care components. Caregiver coverage and its various dimensions are reported significantly higher than in reality, resulting in inaccurate and unreliable reports and outputs from the SIB system.”* (Interview 2, Participants 4).

#### Lack of a verification method to ensure the accuracy of the submitted data and other performance reports in the SIB system

Despite the obligation to validate information and reports received from health centres, no precise process has been defined, and no specific person or unit has been designated in this respect. Unfortunately, health staff record unrealistic data for various reasons, some briefly described, and do not oversee the entry of this data or its subsequent verification. Surprisingly, even though supervisors at various levels of care are aware of these facts, they do not take corrective action. In this corporate culture, hoping to institutionalise an accreditation program and attain the desired level of healthcare quality is impossible.

*“A defined verification mechanism is required to assure the accuracy of the care data entered into the SIB system. These health data will then be transformed into tables, charts, and reports that will serve as the foundation for future health decisions, which is quite concerning.”* (Interview 2, Participants 3).

### Macro-executive infrastructure

#### Lack of accreditation and quality improvement units in the deputy of the Ministry of Health, the University of Medical Sciences, and the country’s health networks

Within the Ministry of Health and Medical Education, there is a need for an organisational body responsible for policy creation, education, and monitoring of the accreditation program’s execution in medical universities and their connected units. However, such a unit does not currently exist. Furthermore, by allocating an organisational unit to accreditation and quality improvement within the central headquarters of health deputies of medical universities and health networks under their management to coordinate matters and provide pertinent specialised advice, the accreditation program in the care system can be institutionalised.

*“Establishing an accredited unit within the Ministry of health can bring together accreditation experts and researchers. It helps to organise them to accomplish macro planning and seek valid scientific evidence, learn from international experiences, train assessors and evaluated persons, and identify and remove barriers to successful accreditation implementation.”* (Interview 1, Participant 6).

#### Insufficient health management specialists and experts in the health care system

Professors, researchers and professionals in health management, particularly health care management, with research and managerial expertise in PHC accreditation, are the best individuals to assist in institutionalising and successfully executing accreditation processes. With an understanding of accreditation’s theoretical and practical foundations, these professionals have a solid capacity to meet the management requirements specified in accreditation standards and metrics. However, healthcare management graduates currently hold the fewest managerial roles.

*“Despite the enormous number of graduates in the health management educational field, no specific program exists to capitalise on the graduates’ scientific and executive talents. The primary issue appears to be the predominance of doctors in the Ministry of Health and, subsequently, in medical universities. The physicians lack specialisation in health administration, particularly accreditation and quality improvement.”* (Interview 2, Participant 5).

#### Lack of participation of international accreditation and quality improvement organisations in the design or implementation of the program

Multiple studies have revealed that, despite being an institution responsible for international accreditation and prominent accreditation organisations worldwide, Iran lacks any educational and consulting opportunities, whether in primary healthcare accreditation or other healthcare-related areas. On the other hand, international organisations and researchers have acquired and amassed information on accreditation from all over the world, resulting in a total of 110 years of experience in accreditation. Iran’s health system will not successfully implement its accreditation program without considering these recorded experiences and the advice services supplied by the organisations.

*“The International Institute for Quality in Health Care (ISQua) now grants licenses to accreditation programs in various countries. They also support international programs and evaluate and grant licenses in three. ISQua also supports publishing educational content and offers advice to countries and health organisations.”* (Interview 1, Participant 3).

#### There is a shortage of accreditation assessors across the country

Evaluators are essential in the certification of health clinics. They extensively analyse health facilities’ compliance with accreditation standards and criteria, and their documented and reasoned reports will be used to grant or refuse accreditation authority to health centres. Iran has no formal structure or procedure for employing assessors in PHC accreditation. Even the Deputy Minister of Health’s hospital care accreditation system lacks a clear and approved framework for selecting and training evaluators. If the unit achieved an acceptable level of performance in this area, it could gain ISQua approval for assessor training.

*“One can only hope for the proper implementation of the processes and activities in the selection and training of accreditation assessors if the requirements defined in the guideline published by ISQua are met. The ministry of health has established the mechanism of selection and training of evaluators to comply with the requirements of the guideline and obtain ISQua approval in this regard.”* (Interview 1, Participant 5).

#### Failure to appoint a competent and non-governmental entity to oversee accreditation with the cooperation of representatives from all relevant parties

A specialised institution comprised of experts in all specialised fields is crucial for implementing the accreditation process and issuing appropriate permissions to health facilities functioning under the country’s PHC system. To be isolated from political influences, this institution must be formed and operate independently on political and organisational levels. The transparency and validity of its decisions must not be questioned. Additionally, it is vital to include representatives from all parties in this institution’s decision-making and policy-making processes to ensure community-oriented actions.

*“The government bodies lack independence in their decision-making and implementation processes, owing mostly to ties with the government. However, a certified institution that is politically and financially self-sufficient is not obligated to manipulate data or ignore health facility inadequacies.”* (Interview 2, Participant 9).

#### Lack of a clearly defined accreditation procedure in Iran

Transparency in work procedures and judgments based on them is crucial for public trust in health centre accreditation, particularly among insurers and service recipients. All associated procedures and actions must be accurately and transparently documented, and evaluators must provide accurate and user-friendly guidelines. Additionally, public confidence in the accreditation licenses can be improved by describing the situation of health facilities across several functional dimensions and publishing documented and reasoned points obtained from the accreditation process.

*“ISQua have developed guidelines on how to define and build accreditation processes, which can be extremely beneficial to countries commencing accreditation programs. Generalisation and a lack of openness in executive process might breed distrust of society, particularly insurance to the final decisions made by accreditation bodies.”* (Interview 3, Participants 3).

#### Excessive system centralisation and a lack of environmental unit authority in the design and execution of health interventions

Although health networks were introduced under the Ministry of Health and Medical Education’s definitions and rules as an independent unit for providing PHC, this independence has not been accomplished in practice. The experience gained in implementing the accreditation program reveals that the authority, independence, and operation of the city’s health facilities and health networks are severely hampered by several burdensome rules and regulations. In such cases, the execution of health interventions to address the deficiencies of health facilities in various functional areas to fulfil the standards and accreditation criteria will be practically limited and weakened.

*“In disbelief, it has been observed that most of the improvement strategies are not feasible for reasons such as restrictions by regulations set by upline managers, economic problems, political restrictions, lack of internal and external cooperation. It would be unfamiliar to talk about promotion and accreditation in a system where managers do not have enough authority to select, relocate, fire, and even punish their subordinates.”* (Interview 1, Participants 1).

#### Failure to approve the program in the form of a law governing its retention in the system

Adopting a promotion program, such as accreditation, as an enforceable law can aid institutionalisation and implementation. In addition to providing an executive guarantee for the program, this would secure its ongoing implementation, diminish the impact of political affairs on it, specify a monitoring and evaluation method, and provide a financial basis for its implementation. International research has shown that programs with legal standing and executive guarantees are more successful and effective in meeting the requirements specified at the heart of accreditation standards and criteria and achieving the desired goals.

*“One of the prerequisites for the successful implementation of accrediting programs is the consent and approval of legal institutions. The critical point here is to develop the appropriate policy and secure long-term financial resources to administer the program, which will assist in its establishment, expansion, and sustainability.”* (Interview 1, Participant 6).

## Discussion

The study was conducted to identify existing challenges in Iran’s PHC accreditation program, which inhibit its successful and effective implementation, using a qualitative approach. The required data were obtained by interviews with contributing managers and staff at various levels of the health system by questions of “what are the challenges/obstacles/shortcomings in successful implementation of Iran’s PHC accreditation program?”

Organisational culture is an idealistic yet practical and effective framework that controls a company and substantially impacts whether organisational goals are achieved. Promoting organisational culture, including quality and safety, teamwork, evidence-based performance, and community-oriented services, is one of the most effective accreditation methods for improving organisational performance. Developing a supportive organizational culture can result in accreditation programs being implemented more quickly and effectively [5]. According to the study’s findings, organisational culture challenges include managers and employees being unaware of the capabilities of quality improvement approaches and evaluations such as accreditation, having a cross-sectional and quick view of accreditation, and impatience in implementing and achieving accreditation objectives. Insufficient collaboration of physical and mental health professionals. A study conducted to ascertain the impediments to implementing a performance-based payment system in PHC in Iran, specifically in the family medicine program, identified the quantitative perspective of managers and employees and a disregard for care quality as two major impediments [12]. In a study on the obstacles facing hospital accreditation programs in Iran, the authors identified cultural barriers, particularly with belief in managers’ and staff’s ability to participate in quality improvement programs and having accurate and factual opinions [13]. Additionally, research aimed at identifying concerns about accreditation in Iranian hospitals revealed a lack of physician and even manager collaboration with the accrediting program [14, 15]. The study designed to ascertain the challenges experienced in implementing the PHC certification program in Lebanon also found organisational culture flaws, most notably low physician participation in the accrediting program [10].

Motivation is the inner energy that propels individuals and organisations to move and strive in a particular direction. Because motivation is the outcome of individual and organisational needs, understanding individual and organisational needs is crucial to generating a desirable level of motivation within an organisation and, as a result, achieving organisational goals. As a result, the design and implementation of incentive systems can significantly impact the successful implementation of quality improvement initiatives, especially accreditation [5]. According to studies, appropriate incentive mechanisms are not defined from an individual standpoint for health workers (financial, occupational, scientific, and spiritual motivators) and an organisational standpoint for health centres and their affiliated units (financial and spiritual motivators). Other barriers in this regard include that many health workers are recruited on temporary contracts, the poor attractiveness of low-income regions in attracting and retaining qualified health managers and personnel, and the dominance of external over internal motives in health managers and employees. One of the most significant challenges has always been the failure to build a practical and objective quality / performance-based payment system and link it to payment mechanisms for work centres, units, and health staff [12]. This problem also exists in the accreditation of Iranian hospitals. Although organizational incentives for successful accreditation programs are partially provided by tying hospital tariffs to the degree of accreditation, appropriate incentives for hospital units, managers, and people have yet to be considered [13]. Other studies have found no financial incentives for employee engagement in the certification program [14, 15].

In Iran, PHC is vast in terms of the number of sectors, labour obligations, and geographical locations. In such instances, one of the primary criteria for the success of health programs is a sufficient number of health workers with executive abilities and capacities [5]. However, the quantity of human resources available to provide health services is often limited, particularly in less privileged areas. Each health worker is expected to carry out activities linked to two or more service units simultaneously and accept a wide range of responsibilities. In addition to the quantity and level of motivation of human resources, their experiences and capabilities, such as having the proper knowledge, attitude, skills, and performance, are crucial to the success of executive programs. One of the issues investigated in this study is that the health worker education system, both at the university level and in the industry, needs to provide managers and health workers with training on quality improvement and accreditation. Health managers have little managerial knowledge or talents, even at the most basic level. Similar studies have identified staff shortages and heavy workloads as significant difficulties in creating quality improvement initiatives.

Furthermore, the main obstacle in developing quality improvement programs is a need for more university and organisational training for managers and staff on accreditation and quality [12]. Iran’s primary challenges in hospital accreditation is a lack of university and organisational training for managers, physicians, and health workers in management, evaluation, accreditation, and quality improvement [13]. According to a similar study, significant workload, a lack of manpower, and their lack of knowledge and abilities in certification-related subjects due to a lack of training are all critical impediments to successfully implementing the accreditation program in Iran [14, 15]. Al-Jardali et al. discovered a scarcity of workers and a double workload as barriers to Lebanon’s PHC accreditation program [12].

In organisations, the information provided in a purposeful, accurate, user-friendly, and timely manner provides a solid and reliable basis for organisational decision-making. It can identify the type and dimensions of the problem, the process of implementing promotional interventions, and the evaluation of learning interventions, among other things. This is especially true when it comes to achieving the intended outcomes. Relevant health indicators must be identified to monitor and analyse the situation, necessary data must be collected and evaluated with a purposeful method, and relevant reports must be delivered to users in a timely, user-friendly, and levelled manner. Finally, the information acquired should be used to make clinical and administrative decisions [5]. As a result of the findings, it can be concluded that the electronic health record systems and statistical panels used in health systems need to be appropriately designed to cover indicators related to health care evaluation. That incorrect data are recorded in them. Although there is no official system for authenticating the data and information acquired, most people cannot report them because no formal procedure exists. According to the findings of a recent study, Iran’s primary healthcare system could be more robust in terms of building an effective and scientific technique for monitoring and evaluating relevant healthcare services. We found a lack of procedures, processes, tools, and manpower available to address the quality issue in PHC assessment, resulting in inefficient information infrastructure and inadequate management of the information generation, storage, and distribution cycle [12].

In addition, efficient accreditation program implementation necessitates suitable executive infrastructure, particularly at the macro level [5]. According to studies, our country has various inadequacies, particularly in PHC certification. These cases include the following: the Ministry of Health is neglecting the field of accreditation and quality improvement in the process of creating structural structures at various levels, the absence of non-governmental organisations to conduct accreditation, the lack of legal approval of accreditation programs at the national level, and the absence of health management experts, the lack of specialised advice from accreditation bodies at the international level, the lack of a transparent and efficient structure for the training of evaluators, as well as the implementation of accreditation, and the limited authority of environmental levels to implement promotional interventions. However, the hospital accreditation program in Iran has been legalised. The work units responsible for evaluation, accreditation, and quality improvement have been expanded from the Ministry of Health to the level of hospitals [13]. The accreditation of PHC is an exception in this case. The absence of an independent national institution to carry out the accreditation process and evaluator training and a lack of adequate and long-term financial resources to support the implementation of the PHC certification program are all significant issues in this regard [13]. One system’s shortcomings are a scarcity of qualified evaluators and a lack of structured training and monitoring programs [12, 14]. Furthermore, it has been suggested that one of Lebanon’s areas for improvement is the prevalence of financial issues for both the accrediting program and the primary health clinics participating in the program [10].

The ISQua collects, analyse and publishes the worldwide accreditation experiences by conducting purposeful studies. These experiences generally reflect in research papers, toolkits, books and practical guidelines. Assessing these experiences, and other study results conducted by other researchers, revealed that the current study findings have notable consistency with previous experiences. On the other hand, the identified challenges in implementing Iran’s accreditation program are similar to other programs developed and implemented in other countries. Therefore the newly developed accreditation programs should learn from the ISQua and other pioneer accreditation programs, in addition to using domestic experts’ and stakeholders’ perspectives, to enhance their capabilities to overcome identified challenges. Likewise, there are notable similarities in required infrastructures and challenges in PHC accreditation fields with other accreditation fields, such as hospital and ambulatory care in various countries [5, 16–21].

As a result, suggestions such as reforming academic and organisational training structures for managers and health workers, providing an adequate quantity and quality of manpower for health centres, developing diverse and sustainable incentive mechanisms for health centres and their staff, targeted strengthening of management and clinical information systems, establishing robust executive mechanisms in the Ministry of Health and beyond with an emphasis on establishing research and development unit, and exploiting the experiences of successful international accreditation programs. Furthermore, designing and conducting another qualitative study to identify more effective suggestion from the view point of health services management and health policy experts is needed. The study’s strengths are that it is among the novel studies in examining the accreditation program’s implementation mechanism in the field of PHC for the first time in Iran and incorporating perspectives from participants in the accreditation pilot program’s implementation. One of the study’s limitations is the small number of individuals who participated in the accrediting pilot program; likewise is the study’s inability to collect expert opinions from throughout the country. Other limitations include the lack of free time for participants to conduct interviews and their fear of catching an infection in group interviews due to the COVID-19 pandemic.

## Conclusion

The fundamental difference between accreditation and other types of evaluation is that accreditation benefits from challenging and updating standards, which results in continuous quality improvement. As a result, achieving the accreditation program’s objectives requires sensible, continuing, thorough, and, most importantly, adequate patience. According to the findings of this study, successfully implementing an accreditation program in the field of PHC in Iran encounters numerous and significant challenges, and overcoming these obstacles requires a concerted effort in designing and implementing promotional interventions. The findings of this study can aid in the practical and continued implementation of the accreditation program and the fulfilment of desired objectives by highlighting challenges and barriers and offering practical methods to overcome them in low and middle-income countries.

## Data Availability

Qualitative data in Persian language is stored in the university of study origin. If any researcher is interested, for valid reason, they may contact the first author (FG), gharibihsa@gmail.com.
